# The contribution of spatial analysis to understanding HIV/TB mortality in children: a structural equation modelling approach

**DOI:** 10.3402/gha.v6i0.19266

**Published:** 2013-01-24

**Authors:** Eustasius Musenge, Penelope Vounatsou, Mark Collinson, Stephen Tollman, Kathleen Kahn

**Affiliations:** 1 MRC/Wits Rural Public Health & Health Transitions Research Unit (Agincourt), School of Public Health, Faculty of Health Sciences, University of the Witwatersrand, Johannesburg, South Africa; 2 Division of Epidemiology and Biostatistics, School of Public Health, Faculty of Health Sciences, University of the Witwatersrand, Johannesburg, South Africa; 3 Swiss Tropical and Public Health Institute, University of Basel, Basel, Switzerland; 4 Centre for Global Health Research, Umeå University, Umeå, Sweden; 5 INDEPTH Network, Accra, Ghana

**Keywords:** HIV/TB, child mortality, conceptual framework, spatial analysis, pathway analysis, South Africa

## Abstract

**Background:**

South Africa accounts for more than a sixth of the global population of people infected with HIV and TB, ranking her highest in HIV/TB co-infection worldwide. Remote areas often bear the greatest burden of morbidity and mortality, yet there are spatial differences within rural settings.

**Objectives:**

The primary aim was to investigate HIV/TB mortality determinants and their spatial distribution in the rural Agincourt sub-district for children aged 1–5 years in 2004. Our secondary aim was to model how the associated factors were interrelated as either underlying or proximate factors of child mortality using pathway analysis based on a Mosley-Chen conceptual framework.

**Methods:**

We conducted a secondary data analysis based on cross-sectional data collected in 2004 from the Agincourt sub-district in rural northeast South Africa. Child HIV/TB death was the outcome measure derived from physician assessed verbal autopsy. Modelling used multiple logit regression models with and without spatial household random effects. Structural equation models were used in modelling the complex relationships between multiple exposures and the outcome (child HIV/TB mortality) as relayed on a conceptual framework.

**Results:**

Fifty-four of 6,692 children aged 1–5 years died of HIV/TB, from a total of 5,084 households. Maternal death had the greatest effect on child HIV/TB mortality (adjusted odds ratio=4.00; 95% confidence interval=1.01–15.80). A protective effect was found in households with better socio-economic status and when the child was older. Spatial models disclosed that the areas which experienced the greatest child HIV/TB mortality were those without any health facility.

**Conclusion:**

Low socio-economic status and maternal deaths impacted indirectly and directly on child mortality, respectively. These factors are major concerns locally and should be used in formulating interventions to reduce child mortality. Spatial prediction maps can guide policy makers to target interventions where they are most needed.

Tuberculosis (TB) and the HIV epidemic are among the top public health priorities especially in sub-Saharan Africa. The sub-Saharan African region, which constitutes 10% of the world's population, is home to more than 67% (22.9 million) of people living with HIV (1). TB, which is preventable and curable, was responsible for 25% of HIV-related deaths in 2009 and infected 33% of those living with HIV. As a result, the World Health Organization (WHO) aims to reduce TB-related deaths by 1 million by 2015 (2). South Africa's population is about 7% [50.6 million (3)] of the sub-Saharan African population, yet accounts for 24.6% (5.63 million of the 22.9 million) of people living with HIV and an estimated 67% of these have TB. South Africa ranks as the country with the highest co-infection rates (1).

Children are affected in two ways: a very high proportion of child deaths (60% in the year 2000) are attributed to the HIV epidemic (4), and approximately 1.9 million children have been orphaned by the HIV epidemic in South Africa (1). The nation at the onset of the 21st century had an estimated 70,000 children [about 25% rate of mother-to-child-transmissions (MTCT)] newly infected, whereas 10 years later only 10,000 [3.5% rate of MTCT (5)], which is indicative of an improved and more effective mother-to-child prevention programme (6). In public health, determining patterns of disease occurrence provides the first steps toward increased understanding of determinants and potentially greater control of the disease (7). Understanding the associated factors of child HIV/TB mortality and how these are geographically distributed is key for effective interventions.

Several determinants of child mortality have been reported over the past three decades. In the 1980s, researchers explored medical, socio-economic, and demographic causes of child mortality (8). In the 1990s, fertility behaviour, nutritional status, breastfeeding and infant feeding, the use of health services by mothers and children, ecological variables, and socio-economic status were studied (9). In the 21st century, the focus has been on HIV exposure, poor maternal health, inadequate infant care, increased exposure to infections, deaths of parents or caregivers, immune system abnormalities, poor nutrition, reduced breastfeeding and antiretroviral treatment exposure (10). These determinants are interrelated and can be combined into a conceptual framework. This combined with spatial aspects forms social ecological models, which are a component of the fast developing systems science that examines multiple effects and interrelatedness of social elements in an environment (11). Analysis of such frameworks can now be handled with the aid of structural equation models also known as pathway analysis.

The motivation for spatial analysis in epidemiological modelling is based on the notion that people living in a household and those in close proximity share similar exposures, which impact on the observed outcomes. In HIV/TB epidemiological research, the focus has largely been on drug development, societal integration, personal nutrition, physical health, and care giving (8–10, 12). Country-wide or region-specific pooled results are often reported, disregarding geographical confounding that commonly exists. Little has been done to interlink factors associated with child HIV/TB mortality or model their impact on areas of residence (households) (13). The few studies implementing spatial analysis have looked across different infectious diseases (14–16), used different study designs (cross-sectional and longitudinal) (17, 18), and considered varying levels of analysis (village, household, and individual) (16, 18, 19). As this is an emerging field, few studies have focused on HIV/TB cause-specific mortality assessing geographical differentials in South Africa over time (19).

The primary aim of this paper was to investigate the cause-specific (HIV/TB) mortality determinants and their spatial (geographical) distribution in the Agincourt sub-district of rural northeast South Africa for children aged 1–5 years in 2004. Our secondary aim was to model how the associated factors were interrelated as either direct (underlying) or indirect (proximal) factors of child HIV/TB mortality using pathway analysis based on the Mosley-Chen conceptual framework that incorporates social, biological, and ecological determinants of child mortality (8).

## Methods

### Agincourt sub-district data and study design

The Agincourt health and socio-demographic surveillance system was established in 1992 in a remote rural sub-district in the Bushbuckridge Municipality of Mpumalanga Province, northeast South Africa near the Mozambique border (20). There are annual census updates that collect socio-demographic information including in-migration and out-migration, mortality, fertility, health, and behavioural data (20).

**Fig. 1:**
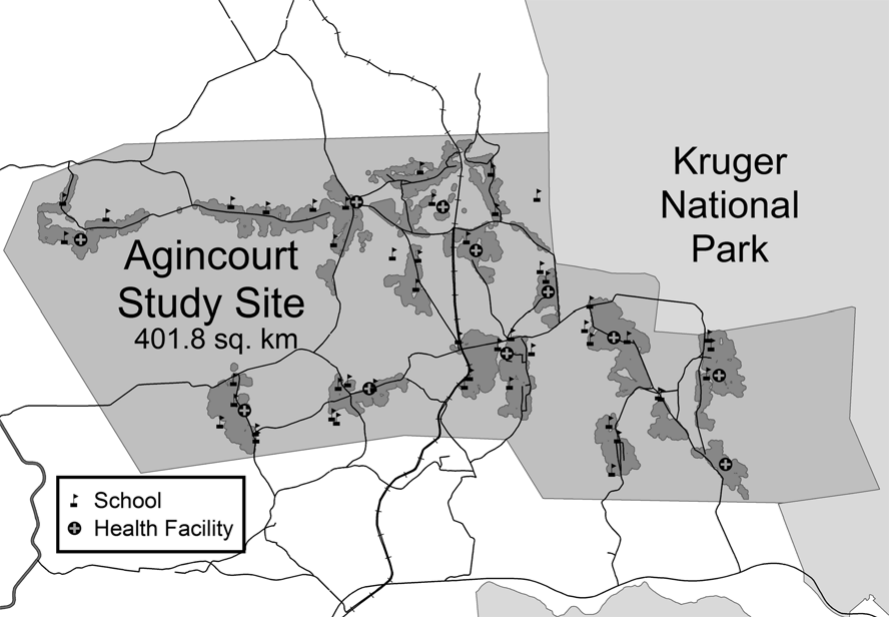
Agincourt sub-district in 2004 showing villages (grey shading) schools (flags), and health facilities (circled grey crosses).

In this study, we extracted data for 2004 describing a cross-sectional study of households in the study site. In that year, the Agincourt health and socio-demographic surveillance system covered a sub-district population of over 70,000 persons living in approximately 12,000 households scattered throughout 21 neighbouring villages. There were several health facilities in the area as shown in Fig. 1.

Cause of death data were obtained through verbal autopsies conducted on every recorded death during the year (21). Trained lay field workers conducted interviews within 1–11 months after a death, with the closest caregiver of the deceased in his/her mother tongue. Two medical practitioners independently determined cause of death; a third diagnosis was sought to resolve discrepancies. Their consensus cause of death was classified according to the WHO International Classification of Diseases 10 (ICD10) (22). The reported signs and symptoms ascertained HIV/TB mortality in children, and in some instances this was substantiated by the mother's cause of death (22). Over 90% of the health and socio-demographic surveillance system (HDSS) households were geo-coded, thus enabling spatial analyses at household level.

### Dependent and independent variables

The study sample included all children aged between 1 and less than 5 years who were registered in the Agincourt HDSS between January and December 2004. The independent variables used were child's gender, nationality and age; mother's age, parity and death; gender of household head, cumulative deaths in household, number of household dwellers, minimum distance to health facility, and household socio-economic status. The latitude and longitude of each household were used to construct the variables for the spatial correlation (random effects), based on the straight line (Euclidean) distances between all pairs of households. The dependent variable was death due to HIV and TB determined by the ICD10 codes A16–A19^1^ for TB and B20–B24^2^ for HIV. The verbal autopsy cannot reliably distinguish HIV and TB due to low sensitivity and specificity; thus, these were combined in our analysis (23).

### Conceptual framework: determinants of child mortality

As a way of interrelating child mortality determinants, we formulated a hybrid conceptual framework for child mortality determinants and disease ecology as proposed by Mosley-Chen and Meade (8, 24). This approach integrates ecological, social, and biological variables in the analysis, with the idea that these determinants operate through a common set of either ‘proximate’ (intermediate or indirect) or ‘underlying’ (direct) variables that impact on HIV/TB child mortality (8, 25–27). Variables are either endogenous (outcome variables) or exogenous (explanatory) or both, and these are modelled using structural equation models. Details of the structural equation model are given in Appendix 1

**Fig. 2:**
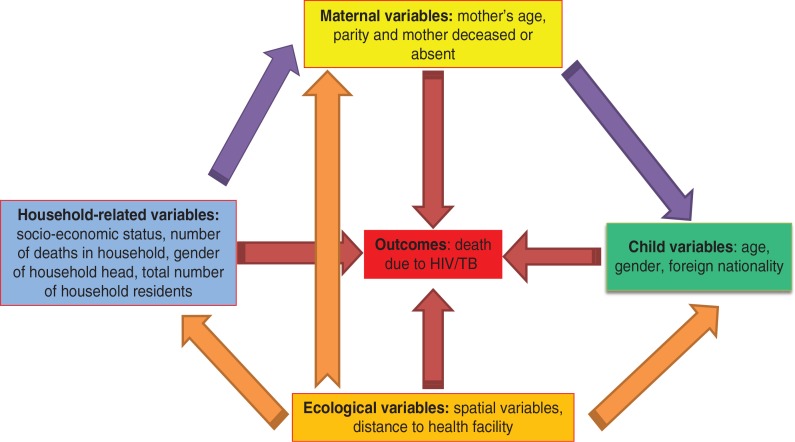
Conceptual framework for modelling childhood HIV/TB-related mortality [adapted from Meade (24)]. The red arrows reflect a direct effect on mortality and the purple (explanatory variables) and orange (spatial random effects) arrows depict an indirect effect.

**Fig. 3:**
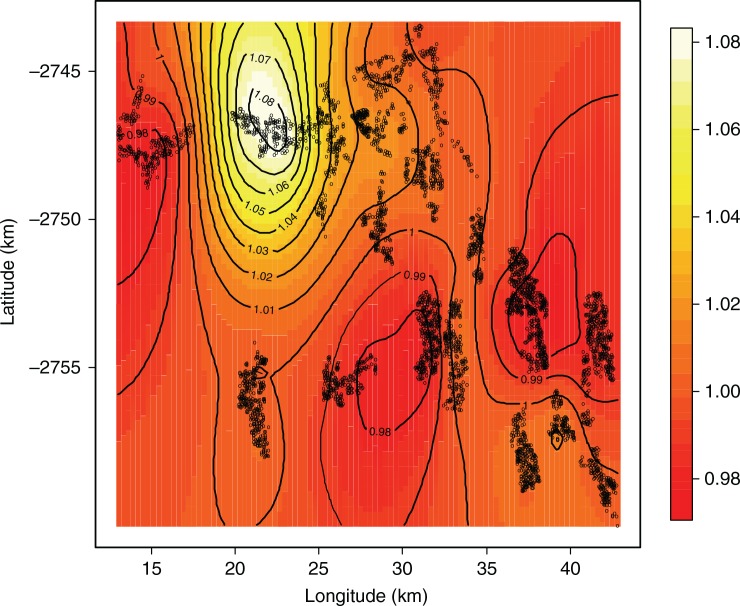
Child HIV/TB mortality: posterior adjusted odds ratio map, Agincourt sub-district, 2004.

We used the observed variables: child specific, mother specific, household related; and unobserved (latent) variables: socio-economic quintiles and spatial (ecological) variables. We tested the pathways shown in Fig. 2, to measure the direct and indirect determinants of child mortality for the Agincourt sub-district in 2004. Two approaches that complemented each other were used in our modelling. Firstly, we modelled the HIV/TB mortality determinants for children aged 1–5 years in 2004 and adjusted for their spatial random effects. In relation to our Fig. 2, we estimated the direct effects pathways (red arrows) from all categories of the explanatory (exogenous) variables controlling for confounders amongst them and their spatial or geographical effects (orange arrows). Results for these are presented in Table 1 and Fig. 3. Lastly, using the significant variables as outcomes (endogenous), we determined the indirect effects (proximal denoted by purple arrows) within and between categories to establish those factors impacting on child HIV/TB mortality.

**Table 1:** Child HIV/TB mortality: descriptive statistics and results of two multiple logit regression analyses, Agincourt sub-district 2004

Variable	Summary number (%)	Univariate logit coefficient (standard error)	Non-spatial multiple logit coefficient (standard error)	Spatial Multiple logit coefficient (standard error)
Age of child in years^1^	2.77±1.20	−0.617 (0.131)**	−0.601 (0.146)**	−0.630 (0.137)**
Gender				
Female	3,379 (50.52)	0	0	0
Male	3,310 (49.48)	0.556 (0.283)*	0.698 (0.303)*	0.641 (0.291)*
				
Child's parents former refugee				
Yes	2,388 (35.71)	0		
No	4,299 (64.29)	−0.137 (0.280)		
				
Mother's age in years^1^	28.70±7.59	−0.003 (0.018)		
Cumulative household deaths^2^	0.68±0.96 (0–6)	0.684 (0.086)**		
Mother deceased by 2004				
No	6,634 (99.13)	0	0	0
Yes	58 (0.87)	1.508 (0.732)*	1.504 (0.867)*	1.386 (0.707)*
Minimum distance to health facility^1^	2.33±1.61	0.078 (0.083)		
Household size^1^	4.88±2.92	−0.011 (0.048)		
				
Gender of household-head				
Female	2,434 (36.37)	0	0	0
Male	4,258 (63.63)	−0.865 (0.418)**	−0.880 (0.302)**	−0.895 (0.30)**
Parity^2^	2.12±1.43 (1–10)	0.085 (0.090)		
				
Socio-economic status quintiles				
Most poor	1,823 (27.24)	0		
Very Poor	1,073 (16.03)	−0.112 (0.393)		
Moderately poor	1,120 (16.74)	−0.516 (0.444)		
Poor	1,316 (19.67)	−0.425 (0.406)		
Least poor	1,360 (20.32)	−0.458 (0.406)		
Non-spatial variance			0.10; 95% BCI (0.001; 0.68)	0.04; 95% BCI (0.001–0.40)
Spatial variance estimate				0.04; 95% CI (0.001–0.28)
Deviance information criteria			457.26±15.56	555.87±11.65

* *p*<0.05

** *p*<0.01.

1 mean±standard deviation

2 mean±standard deviation (range). 95% BCI=95% Bayesian credible interval.

### Statistical methodology

We modelled the child HIV/TB mortality data to find associated factors and determine geographic disparities. Statistical techniques take into account spatial correlations by introducing spatially structured random effects into the model. These are usually based on independent observations. However, with multilevel correlated structures commonly used, maximum likelihood estimation (MLE) methods often underestimate the standard error; thus, the statistical significance is overestimated (28). Analysis was carried out using a geospatial logit regression model, which catered for individual- and household-level random effects (29). Inference was done using classical MLE and Bayesian inference techniques to fit the non-spatial and spatial models, respectively. Bayesian estimation of the many parameters was done using Markov Chain Monte Carlo (MCMC) simulations with the aid of Metropolis Hastings (MH) steps (29). Geospatial prediction maps showing the geographical coordinates, adjusting for child HIV/TB mortality determinants, were used in the analysis.

Data extraction was carried out using Structured Query Language (SQL), data management was done in STATA 10.0 (30), and structural equation modelling was performed in STATA 12.1 (Stata Corporation, College Station, Texas, USA). The Bayesian models were fit using software available in the public domain: BayesX version 2.1 for the spatial modelling and R-cran version 2.12.2 for geospatial mapping using the analysis and mapping packages, maptools, maps, spBayes, and Multilevel B-spline Approximation (MBA) (31, 32).

### Ethical clearance and informed consent

While this study used secondary data, the original study on which it was based was careful to consider ethical issues. Verbal informed consent was obtained when the Agincourt health and socio-demographic update rounds were conducted and also when verbal autopsy data were collected from a close relative of the deceased. To avoid emotionally stressing the interviewee, a culturally accepted period of 1 month was observed before the interview was conducted. Risks of stigmatisation were minimised through special training of fieldworkers on the importance of confidentiality. This work was granted ethical clearance by the University of the Witwatersrand's Committee for Research on Human Subjects (No. 960720 and M081145).

## Results

Of the 6,692 children between 1 and less than 5 years, with a mean age of 2.8 years, 54 HIV/TB-related deaths were recorded in the HDSS in 2004. There were 5,084 fully geo-coded households that recorded children either living or dead. Just over half the children were female (51%), and more than a third (36%) were born to former Mozambican refugees. The mean age of mothers was 29 years, and the mean distance to the nearest health facility was 2.33 km. Table 1 shows the results for the descriptive, univariate, and multivariable logit regression analyses. Maternal death had the greatest effect on child HIV/TB mortality [logit=1.386 (0.707)], equivalent to an adjusted odds ratio (AOR) of 4.00 [95% Bayesian credible interval (BCI)=1.01–15.80] controlling for spatial correlation and keeping all other variables constant.

Multivariable analysis showed three further significant predictors of child mortality in 2004, which were child, maternal, and household specific. Adjusting for spatial correlation and keeping all other variables constant, there were several significant determinants of child HIV/TB mortality. Child-specific differentials showed that boys were almost twice as likely to die compared to girls [logit=0.641 (0.291); AOR=1.89; 95% BCI=1.07–3.36]. As the children got older, there was a protective effect against death; for every year the child aged, the likelihood of death decreased by about 50% [logit=−0.630 (0.137); AOR=0.53; 95% BCI 0.40–0.70]. Male-headed households had a 60% lower risk of child HIV/TB mortality compared to female-headed households [logit=−0.895 (0.30); AOR=0.41; 95% BCI 0.23–0.74].

Spatial disparities can be viewed on maps, which can show unexpected relationships that may have been overlooked in standard regression analysis. We obtained household-specific posterior odds ratios, which were adjusted for child-, maternal-, and household-related variables in the multivariable models. One main hotspot emerged with high mortality in the north, and there were two southerly areas with low mortality. The odds ratio posterior map in Fig. 3 shows the hotspot area between longitude 20° and 25° and latitude −2,750**°** and −2,745**°**. This area is characterised by the following: higher rate of maternal deaths, more male child mortality, lack of a health facility, and being furthest from any health facility as shown in Fig. 1.

**Fig. 4:**
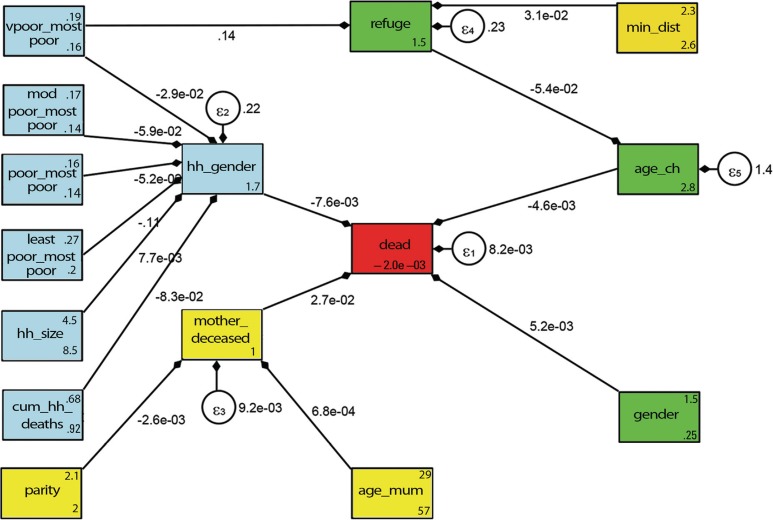
Conceptual framework pathways results for Agincourt sub-district child mortality determinants in 2004. Key for variable names: very poor versus most poor (**vpoor_mostpoor**), moderately poor versus most poor (**modpoor_mostpoor**), least poor versus most poor (**leastpoor_mostpoor**), poor versus most poor (**poor_mostpoor)**, age of child in years (**age_ch**), household size (**hh_size**), cumulative household deaths (**cum_hh_deaths**), gender of household head (**hh_gender**), mother age in years (**age_mum**), mother deceased by 2004 (**mother_deceased**), child born to former refugee parents (**refuge**), gender (**gender**), minimum distance to health facility (**min_dist**) parity (**parity**), child deceased in 2004 (**dead**). Other: the arrows pointing from the exogenous (explanatory) to endogenous (dependent) variables and the error terms (***ɛ***) placed on all five endogenous variables.

The results from the conceptual framework analysis of child HIV/TB mortality are shown in Fig. 4 and Table 2. All of the connected arrows show pathways that were statistically significant whether directly or indirectly. Being male (logit=5.2×10^−3^) and older child age (logit=−4.6×10^−3^) both impacted directly on HIV/TB mortality controlling for potential confounders related to the maternal and households variables. Upon treating male household headship as an exogenous variable, this directly impacted negatively on child HIV/TB deaths (logit=−7.6×10^−3^), adjusting for household variables. Moreover, when male headship was an endogenous variable, the following household variables indirectly impacted mortality: better socio-economic status and larger household size had a protective effect (logit=7.7×10^−3^) and increase in household deaths resulted in greater child HIV/TB mortality (logit=−8.3×10^−2^).

**Table 2:** Direct, indirect and total effects of child HIV/TB mortality in 2004, Agincourt sub-district

	Direct effects on the deceased child as shown in Fig. 2 conceptual framework						
			
	Gender of household-head	Child a former refugee	Child deceased	Mother deceased by 2004	Age of child in years	Indirect effects on child HIV/TB death	Total effects on child HIV/TB death
Very poor vs most poor	−0.029 (0.019)	0.140 (0.015)***				2.6×10^−4^ (1.6×10^−4^)^ns^ !	2.6×10^−4^ (1.6×10^−4^)^ns^
Moderately poor vs most poor	−0.059 (0.020)***					4.5×10^−4^ (2.1×10^−4^)**	4.5×10^−4^ (2.1×10^−4^)**
Poor vs most poor	−0.052 (0.020)***					4.0×10^−4^ (2.0×10^−4^)**	4.0×10^−4^ (2.0×10^−4^)**
Least poor vs most poor	−0.111 (0.018)***					8.4×10^−4^ (3.0 ×10^−4^)***	8.4×10^−4^ (3.0×10^−4^)***
Household size vs most poor	0.008 (0.002)***					−5.9×10^−5^ (2.5×10^−5^)**	−5.9×10^−5^ (2.5×10^−5^)**
Cumulative household deaths	−0.083 (0.006)***					6.4×10^−4^ (2.1×10^−4^)***	6.4×10^−4^ (2.1×10^−4^)***
Minimum distance to health facility		0.031 (0.004)***				7.72×10^−6^ (4.88×10^−6^)^ns^	7.72×10^−6^ (4.88×10^−6^)^ns^
Male household-head			−0.0076 (0.002)***				−0.0076 (0.002)***
Mother deceased by 2004			0.027 (0.012)*				0.027 (0.012)**
Age of child in years			−0.0046 (0.001)***				−0.0046 (0.001)***
Gender			0.0052 (0.0023)**				0.0052 (0.0023)**
Mother's age (years)				0.001 (0.000)***		1.9×10^−5^ (9.99×10^−6^)*	1.9×10–05 (9.99×10^−6^)*
Parity				−0.003 (0.001)**		−7.0×10^−5^ (4.0×10^−5^)^ns^	−7.0×10–05 (4.0×10^−5^)^ns^
Parents former refugee					−0.054 (0.032)*	−2.5×10^−4^ (1.5×10^−4^)*	−2.5×10^−4^ (1.5×10^−4^)*
Intercept	1.719 (0.017)***	1.537 (0.011)***	−0.002 (0.014)	0.995 (0.005)***	2.814 (0.054)***		

* *p<*0.10

** *p<*0.05

*** *p<*0.01

ns ns: not significant

! indirect effects computed as the product along the related pathways, i.e −0.029×−0.0076+0.14×−0.054×−0.0046=2.55×10^−4^≈2.6×10^−**4**^.

Table 2 details the direct pathways for child HIV/TB deaths (arrows directly linked to the red square) and the indirect pathways showing all possible routes for child HIV/TB deaths as shown in Fig. 4. In our analysis, poverty indirectly impacted on HIV/TB child mortality as conferred by two pathways: first, male headship of households (protective), and second, refugee status (a risk) controlling for child's age (as shown on Table 2 foot note). The greatest direct impact on child HIV/TB mortality was maternal death (logit=2.7×10^*−*2^), adjusting for the indirect effects of parity (logit=−2.6×10^*−*3^) and mother's age (logit=6.8×10^*−*4^).

## Discussion

In this study, we showed that HIV/TB child mortality is influenced by a combination of child, maternal, household, and spatial determinants. Over the years 2002–2005, HIV/TB alone accounted for 34% of all-cause mortality among children living in the area (23). While being male was a risk factor for HIV/TB mortality as children got older, irrespective of sex they were less likely to die. A major finding in our study was that maternal death had the greatest direct impact on HIV/TB child deaths. A comparable study in the same area, adjusting for spatial random effects at village level, showed that infants whose mothers had died were at greater risk of death during the first 12 months of life (16).

The main source of TB transmission among children are adults, as shown by a study on a high-risk spatial cluster in South Africa with an incidence three times higher than the WHO levels (17). The most likely cause of HIV infection was mother-to-child transmission. Prevention of mother-to-child transmission (PMTCT), using a single-dose of nevirapine (sdNVP) administered to the child and mother, was found to reduce child mortality by about 9% in South Africa (33). Between 2004 and 2012 the guidelines for PMTCT and ART eligibility have changed. There is also a seven-fold lower (from 25 to 3.5%) rate of infection from mother to child over the period 2000–2010 (5). Kimani-Murage et al. (2009) found that knowledge of child HIV status influenced the manner of care-giving as well as acceptance and up-take of community-wide interventions (31). The mother's survival significantly increases the likelihood of the child's survival, a finding that strengthens the current policy focus on maternal health. In South Africa, a comprehensive three stage process has been set up to improve maternal, foetal, infant, and ultimately child survival through antenatal care, labour and delivery, and postnatal care (34). Reduction of TB morbidity and mortality is the focus of the WHO 2015 goal of zero new TB infections and zero TB-related deaths (2). The positive impact of the PMTCT programme and ART access has also been demonstrated in another rural South Africa site (35).

In Agincourt, female-headed households had a higher child mortality compared to those headed by males. Studies in Tanzania, Zambia, and Limpopo province South Africa showed similar results (36–38). The pathway analysis (Fig. 4) demonstrates that male-headship does not independently lead to lower mortality but is a proxy for better socio-economic status of households. Main breadwinners are usually male with females having fewer opportunities in the labour market and consequently earning less. The absence from the household of a male due to death or permanent out-migration tended to impact negatively on household socio-economic status, leading to 90% greater child mortality odds (39). In other African countries, similar patterns were observed as men temporarily migrate within a country or to neighbouring countries due to either pull or push factors (40–42). HIV/TB deaths have lowered the life expectancy of working men from the area who often “return home to die” from their jobs (21). A recent qualitative study found that a father's unemployment, inability to work, and reluctance to provide support were common factors in HIV-infected households (31). From our pathway analysis (Table 2 and Fig. 4), we observed an indirect impact on child HIV/TB mortality: as the number of household deaths increased, the risk of child deaths was higher. This suggests that households with a greater burden of mortality are more vulnerable socio-economically through loss of breadwinners and high health care and funeral costs. This compromises a family's ability to feed and care for all children, including those who are HIV positive. We also observed that former Mozambicans are at greater risk of child HIV/TB deaths, indicating the need for health care interventions that target these marginalised and vulnerable households.

## Strengths and limitations of the study

The strengths and limitations of this paper are discussed in four categories: classification of exposures and outcomes; local or global relevance of results; confounding and effect modification; and analysis deviance.

A major strength in our observed variables is that the data are consistently updated, corrected, and checked for inconsistencies. Nevertheless, there are potentially two variable-related biases, namely selection and recall bias. Selection bias may occur if permanent out-migrants differ from residents or temporary migrants, which results in incomparable groups. Recall bias on the outcome variable mortality is unlikely as death is a major household event. However, it may affect the quality of the verbal autopsy interview, conducted at least 1 month but up to 11 months after death, and hence the ability of medical experts to assign a likely cause of death.

Our study focused on one sub-district only, but demonstrated geographical differentials in child HIV/TB mortality. There was one main hotspot with highest mortality and a visible downward trend in mortality moving southwards. These patterns are comparable to those found in other studies carried out in the area (16, 19). While many rural areas are not geo-coded to enable direct comparison of trends and patterns, our findings reflect similar patterns in other rural settlements at least in northeast South Africa.

To deal with confounding variables, we used multiple regression and added the spatial random effects in our modelling and also performed pathway analysis. Mediation was tested for in the pathway analysis as we determined factors directly and indirectly related to mortality and malnutrition. However, we were unable to model time varying covariates as this was a cross-sectional study design.

The robustness of our analysis procedures was demonstrated in that after catering for the spatial household random effects, we obtained narrower confidence bands compared to the non-spatial analogue. Therefore, catering for the spatial random effects further improves the model and parameter estimation compared to classical statistical models. Bayesian and pathway analysis combined were able to handle the complex structure of hierarchical data from the Agincourt health and socio-demographic surveillance system. Other advantages of Bayesian modelling include the ability to adjust for spatial confounding, and the ability to handle many parameters and inclusion of prior-known information on the distribution of data.

## Conclusion

The growing field of spatio-temporal epidemiology brings in person, place, and time aspects in the risk factor analysis. Spatial Bayesian models provide better-fitting models compared to non-spatial models. This procedure also enables the drawing of risk maps, which can be used by policy makers to target and develop community-based interventions.

Going forward, further studies investigating trends over time using spatio-temporal Bayesian analysis will strengthen the evidence on associated factors and trends found in this study and demonstrate the value of this analytic approach. Studies comparing deaths due to other causes with those for HIV/TB in children would add policy value. Similarly, adopting a secondary analysis of a cohort study design would have enabled temporality to be investigated in greater detail and survival analysis to be undertaken. Moreover, a combination of qualitative and quantitative analysis methods is needed to understand the proximal and distal factors that influence child HIV/TB mortality. Findings that can be used for national policy would require sentinel sites located strategically across the country.

## Footnotes

1 A16=Respiratory tuberculosis; not confirmed bacteriologically or histologically; A17=Tuberculosis of nervous system; A18=Tuberculosis of other organs; A19=Miliary tuberculosis.

## Footnotes

2 B20=Human immunodeficiency virus (HIV) disease resulting in infectious and parasitic diseases; B21=Human immunodeficiency virus (HIV) disease resulting in malignant neoplasms; B22=Human immunodeficiency virus (HIV) disease resulting in other specified diseases; B23=Human immunodeficiency virus (HIV) disease resulting in other conditions; B24=Unspecified human immunodeficiency virus (HIV) disease.
